# TiO_2_ Nanosols Applied Directly on Textiles Using Different Purification Treatments

**DOI:** 10.3390/ma8115437

**Published:** 2015-11-24

**Authors:** Simona Ortelli, Anna Luisa Costa, Michele Dondi

**Affiliations:** Institute of Science and Technology for Ceramics—Italian National Research Council, Via Granarolo 64, Faenza (RA) I-48018, Italy; simona.ortelli@istec.cnr.it (S.O.); michele.dondi@istec.cnr.it (M.D.)

**Keywords:** nano-TiO_2_, purification process, anion exchange resin, photocatalytic performance

## Abstract

Self-cleaning applications using TiO_2_ coatings on various supporting media have been attracting increasing interest in recent years. This work discusses the issue of self-cleaning textile production on an industrial scale. A method for producing self-cleaning textiles starting from a commercial colloidal nanosuspension (nanosol) of TiO_2_ is described. Three different treatments were developed for purifying and neutralizing the commercial TiO_2_ nanosol: washing by ultrafiltration; purifying with an anion exchange resin; and neutralizing in an aqueous solution of ammonium bicarbonate. The different purified TiO_2_ nanosols were characterized in terms of particle size distribution (using dynamic light scattering), electrical conductivity, and ζ potential (using electrophoretic light scattering). The TiO_2_-coated textiles’ functional properties were judged on their photodegradation of rhodamine B (RhB), used as a stain model. The photocatalytic performance of the differently treated TiO_2_-coated textiles was compared, revealing the advantages of purification with an anion exchange resin. The study demonstrated the feasibility of applying commercial TiO_2_ nanosol directly on textile surfaces, overcoming problems of existing methods that limit the industrial scalability of the process.

## 1. Introduction

Textile finishing involving the application of inorganic colloidal nanosuspensions (nanosols) can give rise to new fabrics in which the properties of inorganic nanoparticles are transferred to the textiles’ surface [1]. Inorganic nanostructured coatings have a good affinity for fabrics and extend the durability of their function by comparison with conventional methods used to impart various properties to textiles. Their tiny particle size (nanoparticles) also makes them transparent to visible light so their presence does not alter the fabric’s color, “hand” and “breathability”. Textile finishes obtained by applying nanosols can be used to transfer several functional properties to a fabric. For example, Shi, *et al.* [2] prepared a water-repellent (hydrophobic) coating for cotton textiles from fluorinated diblock copolymers. Xu, *et al.* [3] used reactive magnetron sputtering to deposit nanoscale TiO_2_ films on the surface of polyester (PET) nonwovens to obtain antistatic materials. Chattopadhyay and Patel [4] reported manufacturing cotton textiles coated with nano-zinc for its antimicrobial properties. Bozzi, *et al.* [5] demonstrated that red wine and coffee stains were photo-discolored and mineralized on textiles coated with nano-TiO_2_ for its self-cleaning properties. In fact, nanostructured TiO_2_ anatase-based coatings have excellent photocatalytic properties, and are widely used in textile stain removal processes under UV irradiation [6,7]. A straightforward and economical way to make textiles with photocatalytic properties deriving from the application of nanosols (a process called “ceramization”, involving a ceramic nanosol such as TiO_2_) is the dip-pad-dry-cure method [8,9,10,11]. In view of a technological transfer to an industrial-scale production of self-cleaning textiles, the method used to apply a commercial TiO_2_ nanosol to textiles needs to be simple. Such a method is described in the present work: it focuses on purifying and neutralizing the commercial TiO_2_ nanosol in order to expand its applicability and improve the photocatalytic performance of the end product. Three different treatments were developed: washing by ultrafiltration, purifying with an anion exchange resin; and neutralizing in an aqueous solution of ammonium bicarbonate. A fundamental aspect to consider is the possibility of changes to physicochemical properties such as pH, surface charge and conductivity as a result of these treatments. This could lead to agglomeration, aggregation or coagulation problems in nanosuspensions, so it is essential to avoid any colloidal destabilization [12,13]. The traditional ultrafiltration method [14,15], already used in our previous works [16,17], was compared here with more innovative approaches involving purification with an anion exchange resin and neutralization after depositing the nano-TiO_2_ coating. Purified and neutralized samples of TiO_2_ nanosol were applied directly on the textile using the dip-pad-dry-cure method. The photo-discoloration of rhodamine B (RhB), used as a stain model, was assessed on untreated and treated textiles and the photocatalytic performance of the differently-treated TiO_2_ coatings on the textile were compared.

## 2. Experimental

### 2.1. Materials

TiO_2_ nanosol (NAMA41, 6 wt %), called TAC, was purchased from Colorobbia (Sovigliana, Vinci (FI), Italy). The commercial nanosol was diluted with deionized water to 3 wt %. A soft furnishing fabric was used in this study with a specific weight of 360 g/m^2^ and a composition of 62% cotton and 38% polyester. The ammonium bicarbonate (purity ≥99.0%), rhodamine B (dye content ∼95%) target dye, and Dowex**^®^** 66 anion exchange resin were purchased from Sigma Aldrich (Milano, Italy).

### 2.2. Methods

The commercial TiO_2_ nanosol (TAC) could not be used as purchased because of its very low pH and very high conductivity (Table 1). The purification treatments were absolutely necessary for two main reasons: (1) the textile substrate is damaged if the acidity falls below pH 3.5 due to acid-catalyzed oxidation phenomena occurring at high curing temperatures; and (2) any residual byproducts of synthesis in the commercial TiO_2_ nanosol could significantly reduce its photocatalytic activity.

The three different treatments applied to the TAC nanosol were:

1. washing by ultrafiltration (TACF);
2. purification with an anion exchange resin (TACR);
3. neutralization of the TAC-coated textile (TACBIC).

They are described in detail below.

**Table 1:** Physicochemical characteristics of TiO_2_ nanosol samples.

Sample	Nominal pH	pH *	D50_DLS_ (nm)	Electrical Conductivity (mS/cm)	pH_i.e.p._
TAC	1.5	2.9	36	1.18	7.09
TACF	4.0	3.3	42	0.25	6.92
TACR	4.5	4.2	94	0.05	6.91
TACBIC	–	5.0 **	–	–	–

* pH measurement of nanosol (0.1 wt % TiO_2_ concentration); ** pH measurement onto textile surface.

#### 2.2.1. Washing by Ultrafiltration (TACF)

Ultrafiltration was carried out using a solvent-resistant stirred cell (Merck Millipore, Vimodrone (MI), Italy) and a polymer membrane with a pore size of 100 kDalton that enabled the TiO_2_ nanoparticles to be retained, thereby increasing the pH while the byproducts of synthesis were removed. The vessel was refilled with water several times until the pH was 4.0. The ultrafiltered sample (TACF) was so obtained.

#### 2.2.2. Purification with an Anion Exchange Resin (TACR)

This process involved adding a weak anion exchange resin to the TiO_2_ nanosol. The resin was able to sequester Cl^−^ ions and release OH^−^ ions with a consequent increase in pH. Once the required pH value had been reached, the resin was removed by simple separation. The pH obtained in the purified sample (TACR) was 4.5.

#### 2.2.3. Neutralization of the TAC-Coated Textile (TACBIC)

This treatment was performed directly on the TAC-coated textile before curing (TACBIC). The process consisted in applying the commercial TiO_2_ nanosol (TAC) on the textile using the dip-pad-dry-cure method. Then, an aqueous solution (0.5 M) of ammonium bicarbonate (NH_4_HCO_3_) was deposited on the TAC-coated textile using a manual spray-coating technique to neutralize the acidity of the commercial TiO_2_ nanosol.

### 2.3. Dip-Pad-Dry-Cure Method

**Figure 1:**
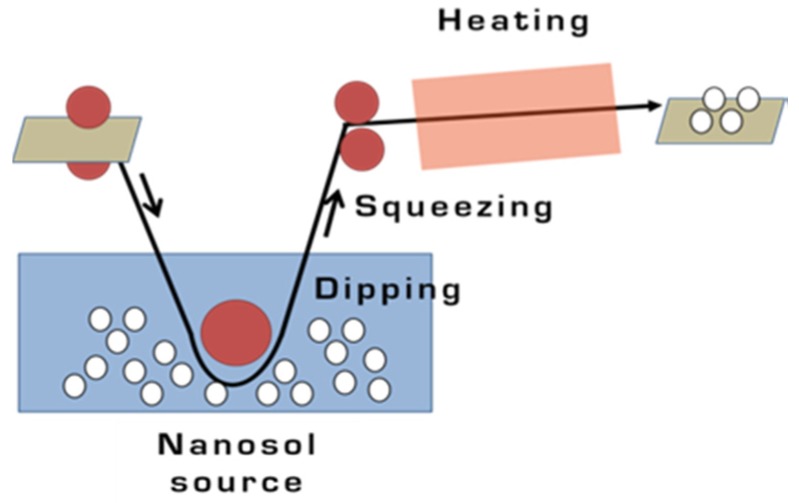
Schematic representation of the dip-pad-dry-cure method.

Fabric samples were washed in an ultrasound bath for 30 min (15 min with soap and water, and 15 min with water alone). The fabric samples thus prepared were dipped in the titania nanosol (3 wt %) and left to soak for 3 min, then passed through a two-roller laboratory padder, oven dried at 100 °C, cured for 10 min at 130 °C, and finally washed in water in an ultrasound bath for 15 min to remove any nanoparticles not physicochemically adsorbed onto the surface. This dip-pad-dry-cure method is illustrated in Figure 1.

### 2.4. Characterization of TiO_2_ Nanosols

The phase composition of the commercial TiO_2_ was ascertained by X-ray diffraction (XRD). The diffraction patterns were obtained directly on the TiO_2_-based nanosols (TAC, TACF and TACR) using a Bragg-Brentano diffractometer (Bruker D8 Advance, Karlsruhe, Germany) operating in a θ/2θ configuration, with an X-Celeretor detector LynkEye (20°–70°, 2θ range, 0.02 step size, 0.5 s per step). The particle size distribution of the nanosols (0.01 wt %) was measured by dynamic light scattering (DLS) through a Zetasizer Nanoseries (Malvern Instruments, Malvern, UK). This technique provides the hydrodynamic diameter of suspended particles, expressed as D50, which is the value of the particle diameter at 50% of the cumulative distribution. The electrical conductivity of the three nanosols (0.1 wt %) was measured with a conductometer (AMEL 134, AMEL, Milano, Italy). Their ζ potential was examined using electrophoretic light scattering (ELS) (Zetasizer Nanoseries—Malvern Instruments, Malvern, UK). The instrument used has an automatic titrating system for measuring the ζ potential of nanosols as a function of their pH (experimental uncertainty: 1 mV for ζ potential and 0.2 for pH). The measurements were performed on sols at low concentrations (0.1 wt %) to prevent precipitation due to pH changes. The titration was done by adding 0.01 M KOH solution. Three measurements were obtained for each sample and the average ζ potential values were considered.

### 2.5. Textile Characterization

The presence of the coating and the amount of nano-titania absorbed by the fabrics was established from the weight difference using a burn-out technique: 0.5 g of sample was burnt at 800 °C and the residual titania was expressed as a *w*/*w* percentage of the TiO_2_-coated fabric.

### 2.6. Photocatalytic Measurements

The pristine fabric sample and titania-coated samples were stained with 0.2 mL droplets of an aqueous solution of rhodamine B (0.07 g/L), chosen as a stain model.

After staining, the samples were irradiated with UV at an intensity of 9 W/cm^2^ (Osram ULTRA-Vitalux lamp, Munich, Germany). The lamp was switched on 30 min before starting the photocatalytic test to stabilize the power of its emission spectrum. The distance between the sample and the lamp was kept constant at 25 cm. To assess the degree of discoloration, the samples underwent colorimetric measurements before and after UV exposure. All colorimetric measurements were performed with a DRS (Miniscan MSXP4000, Hunter Lab, Reston, VA, USA) in the 400–700 nm range (illuminant D65, observer 10°). Color was expressed using CIELab parameters: brightness (L*: 100 = white 0 = black) and chroma (a*: red +, green −; b*: yellow +, blue −). Photocatalytic performance was judged by assessing the amount of discoloration, expressed in terms of efficiency (%). In particular, the color difference (∆E*) between the samples before and after exposure was calculated as follows:

ΔE* = [(ΔL*)^2^ + (Δa*)^2^ + (Δb*)^2^]^1/2^ (1)

and referred to the pristine sample by subtracting the background color of the fabric. Our color difference (ΔE*%) assessment method is explained in detail in our previous studies [17,18], and correlated with photodegradation efficiency.

## 3. Results and Discussion

### 3.1. Characterization of TiO_2_ Nanosols

**Figure 2:**
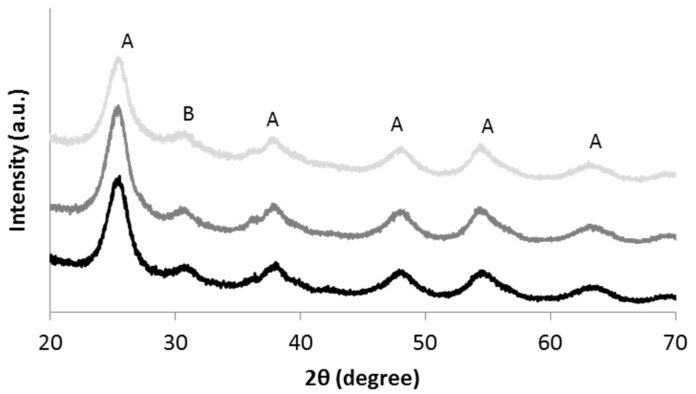
XRD diffractograms of TAC (light gray), TACF (medium gray) and TACR (black); (A = anatase; B = brookite).

Figure 2 shows the XRD diffractograms of the different samples. The results confirmed that pH changes induced by the purification treatments did not affect the crystalline phases. The main phase detected was anatase (JCPDS card n. 21-1272) with a small amount of brookite (JCPDS card n. 29-1360). The broad peaks typical of nano-sized crystallites were detected for all samples.

The three different treatments on the commercial TiO_2_ nanosol (TAC) enabled an increase in pH and particle size, and a decrease in conductivity (Table 1).

The DLS data showed an increase in particle diameter in the TACF and TACR samples, caused by a greater degree of agglomeration, and an associated increase in pH.

**Figure 3:**
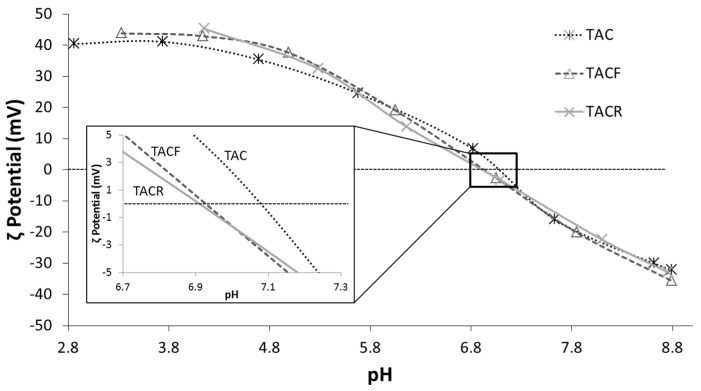
ζ potential *vs*. pH of TAC, TACF and TACR samples.

**Figure 4:**
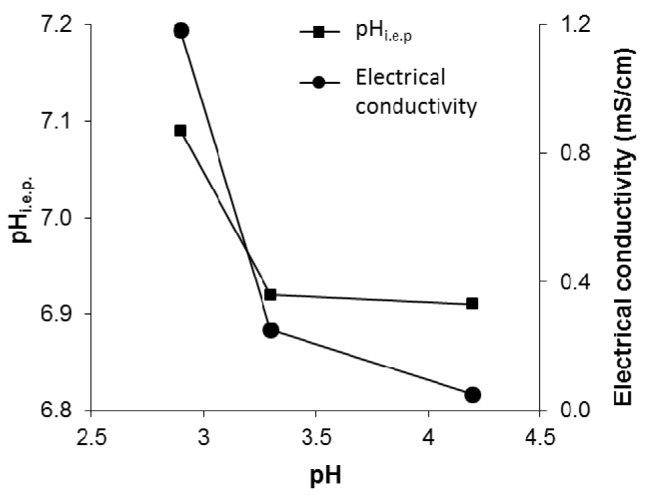
Influence of pH on pH_i.e.p._ and electrical conductivity.

Figure 3 shows the ζ potential *vs*. the pH for the three different purified TiO_2_ nanosols, from which we can identify the pH_i.e.p._ at the crossover point from positive to negative ζ potential values. The three curves were quite similar, but a slight shift in pH_i.e.p._ towards an acid pH from the TAC sample to the TACR sample was apparent, *i.e.*, the baseline pH increased. These data are in agreement with previous reports [16] and indicate a higher surface acidity due to a higher pH and increased agglomeration, as we can see from the hydrodynamic diameters given in Table 1. On the grounds of our previous works [16,18], an increase in surface acidity was expected to coincide with an increase in surface hydrophilicity. The pH values were consistent with the conductivity data (Table 1). The high conductivity value for the TAC sample was associated with the presence of residual byproducts of synthesis. The successful purification of the commercial TiO_2_ nanosol (TAC) was demonstrated by the higher pH and lower conductivity of the TACF and TACR samples. There was a good correspondence between the shift in pH_i.e.p._ towards a more acid pH and the drop in conductivity as a function of pH, as shown in Figure 4. In particular, the TACR sample showed a very low conductivity, demonstrating that purification with an anion exchange resin was more efficient in removing byproducts. The increase in pH achieved with the ultrafiltration process never exceeded 4.0, whereas purification with an anion exchange resin has no maximum limit. At pH > 4.5, however, the TiO_2_ nanosol underwent colloidal destabilization.

The anion resin exchange efficiently removed byproducts, and Cl^−^ anions in particular. The exchange reaction is as follows:

R-NH_3_OH + Cl^−^ → R-NH_3_Cl + OH^−^ (2)

It is easy to regenerate the anion exchange resin by washing it with a highly-concentrated NaOH solution [19,20], so purification processes based on ion resin exchange are economical and easily scalable, as many papers focusing on large-scale ion exchange processes have demonstrated [21,22,23,24,25].

### 3.2. Characterization of Textiles

A homogeneous coating of nano-TiO_2_ was obtained, as confirmed by the burn-out test. A white TiO_2_ powder agglomerate with an appearance perfectly mimicking the texture of the fabric’s fibers was achieved. The amount of TiO_2_ (about 3 wt %) is consistent with the tested capacity of cotton-polyester fabrics to adsorb an amount of sol equating to their weight. In particular, the residual TiO_2_ after burn-out was 3.0, 3.4, 3.5 and 3.0 wt % in the TAC, TACF, TACR and TACBIC, respectively.

**Figure 5:**
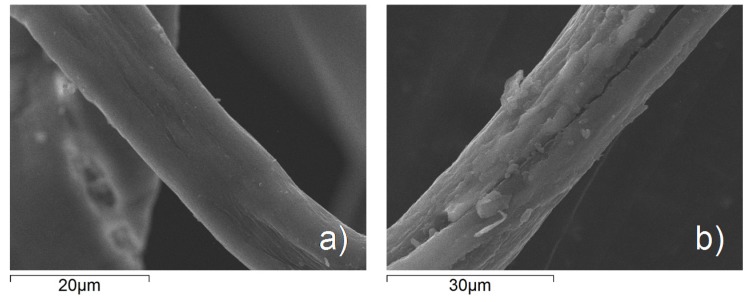
SEM micrographs of: (**a**) an uncoated fabric fiber and (**b**) a fabric fiber coated with the TACF nanosol.

SEM analysis (Figure 5) showed the changes in surface morphology induced by the presence of TiO_2_ nanoparticles confirming the formation of a homogeneous nano-TiO_2_ coating on the fabric’s surface. Unlike the smooth texture of the uncoated fiber (Figure 4a), the fibers in the TACF-coated fabric (Figure 4b) showed a certain surface roughness due to the thin layer of TiO_2_ adhering to the textile substrate.

### 3.3. Photocatalytic Measurements

**Figure 6:**
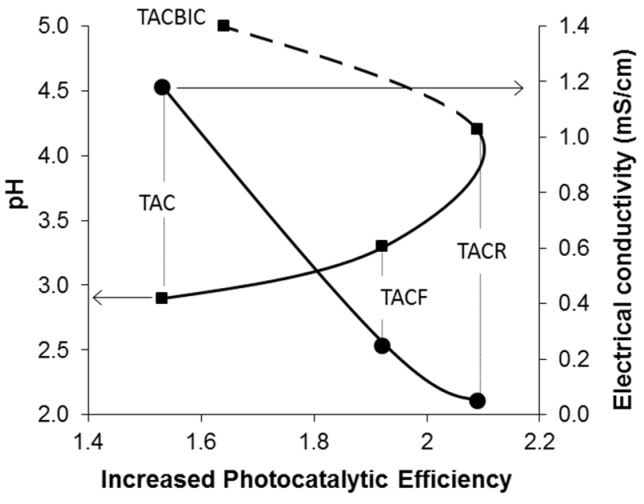
Increase in photocatalytic efficiency as a function of pH (▪) and electrical conductivity (●).

Photocatalytic activity was assessed in terms of discoloration of a stain caused by an aqueous solution of rhodamine B on the pristine fabric and the samples coated with differently-treated nano-TiO_2_. The photocatalytic results are expressed in terms of photochemical efficiency values, calculated taking the uncoated fabric’s photocatalytic efficiency for reference (Figure 6). The various treatments induced an improvement in the fabric’s photocatalytic activity with the following trend: TACR >TACF >TACBIC > TAC. The results of the photocatalytic measurements are summarized in Table 2.

**Table 2:** Photocatalytic results obtained on TiO_2_-coated fabric samples.

Sample	Photocatalytic Efficiency (%)	Increase in Photocatalytic Efficiency *
TAC	68.5	1.54
TACF	84.3	1.90
TACR	92.5	2.08
TACBIC	73.2	1.65

* *vis-à-vis* the uncoated fabric sample (photocatalytic efficiency: 44.4%).

As expected, the presence of residual byproducts of synthesis in commercial TiO_2_ nanosol (TAC) gave rise to a low photocatalytic efficiency. Even after post-neutralization (which increased the pH on the surface of the TiO_2_-coated fabric), the photocatalytic performance of the TACBIC sample was only slightly better than that of the TAC sample. It was probably the presence of residual byproducts synthesis in the sample that impaired its photocatalytic activity. Better results were obtained with treatments applied directly to the nanosol (the TACF and TACR samples). Despite a strong degree of agglomeration in the TACR sample, the purification treatment with an anion exchange resin produced the best performance. The main contributor to the improvement in photocatalytic performance was therefore the removal of residual byproducts of synthesis, as demonstrated by the treated sample’s low conductivity and high pH, indicative of a high surface acidity and a consequently high hydrophilicity. The photocatalytic results revealed the importance of using a purified nanosol in order to obtain a good end product performance. The neutralization treatment proved less effective in improving photocatalytic performance than the purification treatment (to remove byproducts), as the weak photoreactivity of the TACBIC-coated fabrics demonstrates.

## 4. Conclusions

The present work describes a method for applying commercial TiO_2_ nanosol directly on textiles with stabilized characteristics. Three different nanosol purification/neutralization treatments were tested and found fundamental to the success of the self-cleaning textile application.

The physicochemical properties of the differently-treated TiO_2_ nanosols showed that a low conductivity and a high pH (corresponding to a high surface acidity) enabled fabrics with high hydrophilic properties and a good photocatalytic performance to be obtained.

Based on the evidence emerging from our photocatalytic experiments, purification to remove byproducts was more effective than a neutralization treatment. In fact, a purification process involving the use of an anion exchange resin proved the most effective treatment. The easy scalability of this process, and the opportunity to control the TiO_2_ nanosols’ physicochemical properties (pH and conductivity) make this method very promising for the industrialization of self-cleaning textile applications.
